# miR-27a attenuates adipogenesis and promotes osteogenesis in steroid-induced rat BMSCs by targeting PPARγ and GREM1

**DOI:** 10.1038/srep38491

**Published:** 2016-12-02

**Authors:** Chenxi Gu, Yan Xu, Shanfeng Zhang, Hongya Guan, Shi Song, Xiuli Wang, Yisheng Wang, Yuebai Li, Guoqiang Zhao

**Affiliations:** 1 Department of Orthopedic Surgery, The First Affiliated Hospital of Zhengzhou University, No. 1 Jianshe East Road, Zhengzhou 450052, China; 2 Department of Biochemistry and Molecular Biology, School of Basic Medical Sciences, Zhengzhou University, No. 100 Kexue Road, Zhengzhou 450001, China; 3 Department of Microbiology and Immunology, School of Basic Medical Sciences, Zhengzhou University, No. 100 Kexue Road, Zhengzhou 450001, China

## Abstract

The imbalance between adipogenic and osteogenic differentiation in bone marrow mesenchymal stem cells (BMSCs) plays a significant role in the pathogenesis of steroid-induced osteonecrosis of the femoral head (ONFH). Several microRNAs (miRNAs) are involved in regulating adipogenesis and osteogenesis. In this study, we established a steroid-induced ONFH rat model to identify the potential relevant miRNAs. We identified 9 up-regulated and 28 down-regulated miRNAs in the ONFH rat model. Of these, miR-27a was down-regulated and negatively correlated with peroxisome proliferator-activated receptor gamma (PPARγ) and gremlin 1 (GREM1) expression. Further studies confirmed that PPARγ and GREM1 were direct targets of miRNA-27a. Additionally, adipogenic differentiation was enhanced by miR-27a down-regulation, whereas miRNA-27a up-regulation attenuated adipogenesis and promoted osteogenesis in steroid-induced rat BMSCs. Moreover, miRNA-27a up-regulation had a stronger effect on adipogenic and osteogenic differentiation in steroid-induced rat BMSCs than si-PPARγ and si-GREM1. In conclusion, we identified 37 differentially expressed miRNAs in the steroid-induced ONFH model, of which miR-27a was down-regulated. Our results showed that miR-27a up-regulation could inhibit adipogenesis and promote osteogenesis by directly targeting PPARγ and GREM1. Thus, miR-27a is likely a key regulator of adipogenesis in steroid-induced BMSCs and a potential therapeutic target for ONFH treatment.

Osteonecrosis of the femoral head (ONFH) is one of the most common orthopedic diseases, which can be divided into traumatic ONFH and non-traumatic ONFH. While traumatic ONFH generally occurs after trauma, the causes of non-traumatic ONFH are complex^1^,^2^, including abnormal circulating functions, alcohol addiction and steroid treatment^3^, of which the wide clinical application of steroids has made steroid-induced ONFH the most common type. Several hypotheses have been developed regarding the mechanism for non-traumatic ONFH, including intravascular coagulation, fat embolism, osteoporosis and steatosis of osteocytes^4^,^5^,^6^,^7^, in which the adipogenic differentiation of bone marrow-derived mesenchymal stem cells (BMSCs) has been widely accepted^8^.

BMSCs are adult fibroblast-like multipotent cells that are usually found in the bone marrow^9^. These cells demonstrate self-renewal properties and can differentiate into diverse fates, including adipocytes, osteoblasts and vascular endothelial cells, both *in vitro* and *in vivo*^10^,^11^. *In vivo* regulation of BMSC differentiation plays a significant role in many physical and pathological processes. For instance, a study in rabbits showed that after exposure to a high dose of steroids, an increased number of adipocytes were detected in the femoral head at the same time as liver steatosis, suggesting that the adipocytes were generated locally instead of being derived from the circulation. Further research showed that the relative expression of the gene encoding peroxisome proliferator-activated receptor gamma (PPARγ) was rapidly increased in BMSCs from the femoral head after steroid treatment, whereas the expression of the osteogenic gene Cbfal/Runx2 decreased. In addition, triglyceride accumulation was observed in steroid-treated BMSCs, suggesting that steroids promote the adipogenic differentiation of BMSCs^12^. Recent research suggests that increased adipocytic differentiation of BMSCs and distorted bone formation in the bone microenvironment are important causes of ONFH^13^, and several pathways (e.g., the Wnt-dependent pathway) are responsible for the switch in BMSC fate between osteoblasts and adipocytes^14^. Thus, to promote osteogenesis while inhibit adipogenesis may serve as an important strategy in treating steroid-induced ONFH.

According to a recent study, a small interfering RNA (siRNA) targeting PPARγ delivered by an adenovirus vector significantly inhibited the adipogenic differentiation of BMSCs in steroid-induced rabbits, thus maintaining osteogenesis and exhibiting a preventive effect on ONFH^15^. Indeed, the combined strategy of both adipogenesis inhibition and osteogenesis promotion may be more efficient for the treatment of ONFH. MicroRNAs (miRNAs) can regulate gene expression by targeting mRNAs and usually have multiple targets; certain miRNAs may be involved in both adipogenesis and osteogenesis and have potential roles in the development of steroid-induced ONFH^16^.

Some studies have demonstrated that miR-27a could target PPARγ mRNA, which was directly associated with adipogenesis^17^,^18^,^19^,^20^. Besides, our miRNA microarray analysis and further bioinformatics analysis indicated that miR-27a was probably involved in the process of adipogenesis and osteogenesis, and had interactions with PPARγ and GREM1 (a gene relevant with osteogenesis). Additionally, our previous study showed that miR-27a could regulate osteogenic and adipogenic differentiation. Hence, in the present study, we concentrated on detecting the role of miR-27a in the adipogenesis and osteogenesis of steroid-induced rat BMSCs and exploring the preliminary mechanisms contributing to its involvement.

## Results

### miR-27a was down-regulated, while PPARγ and GREM1 were up-regulated in the steroid-induced ONFH rat model

**Figure 1:**
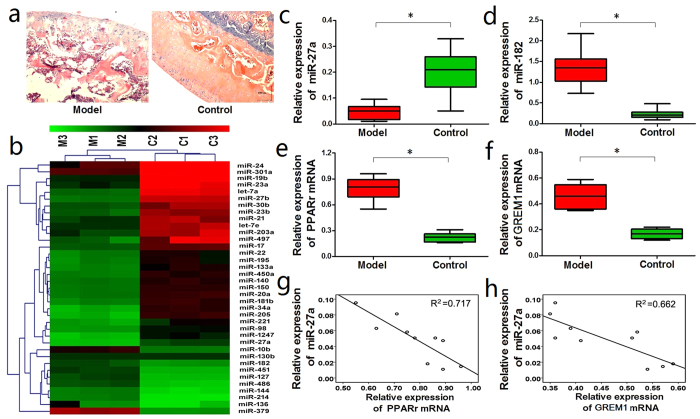
miR-27a was down-regulated while PPARγ and GREM1 were up-regulated in the steroid-induced ONFH rat model. (**a**) HE staining showed that the bone trabecula was thinner, sparser, smaller, and more commonly fractured and disordered in the Model group compared with the Control group. (**b**) The microarray analysis identified 37 differentially expressed miRNAs in the Model group. (**c**) qRT-PCR detected a significant down-regulation of miR-27a in the Model group. (**d**) qRT-PCR detected the up-regulation of miR-182 in the Model group. (**e, f**) qRT-PCR detected a significant up-regulation of the PPARγ and GREM1 mRNAs in the Model group. (**g, h**) The PPARγ and GREM1 mRNA levels were negatively correlated with miR-27a expression. ^*^P < 0.05.

In the Model group, hematoxylin and eosin (HE) staining of the femoral heads showed that the bone trabecula was thinner, sparser, smaller, and more commonly fractured and disordered compared with the Control group (Fig. 1a).

The miRNA microarray analysis identified 37 differentially expressed miRNAs (9 up-regulated and 28 down-regulated) in the femoral heads from the Model group, which displayed at least a 3-fold change compared with the miRNA expression profiles in the Control group (Table 1, Fig. 1b). qRT-PCR was performed to determine the expression levels of miR-27a and miR-182, and to verify the microarray analysis. Corroborative results were obtained, showing that miR-27a was significantly down-regulated while miR-182 was obviously up-regulated in the Model group (Fig. 1c,d).

PPARγ and gremlin 1 (GREM1), two relevant genes involved in adipogenic and osteogenic differentiation, were also examined by qRT-PCR. Both the PPARγ and GREM1 mRNAs were significantly up-regulated in the femoral heads from the Model group compared with the Control group (Fig. 1e,f).

Because miR-27a was down-regulated in the Model group while PPARγ and GREM1 were both up-regulated, we next performed a correlation analysis, which showed that PPARγ and GREM1 were both negatively correlated with miR-27a expression (Fig. 1g,h).

### PPARγ and GREM1 were direct targets of miRNA-27a

**Figure 2:**
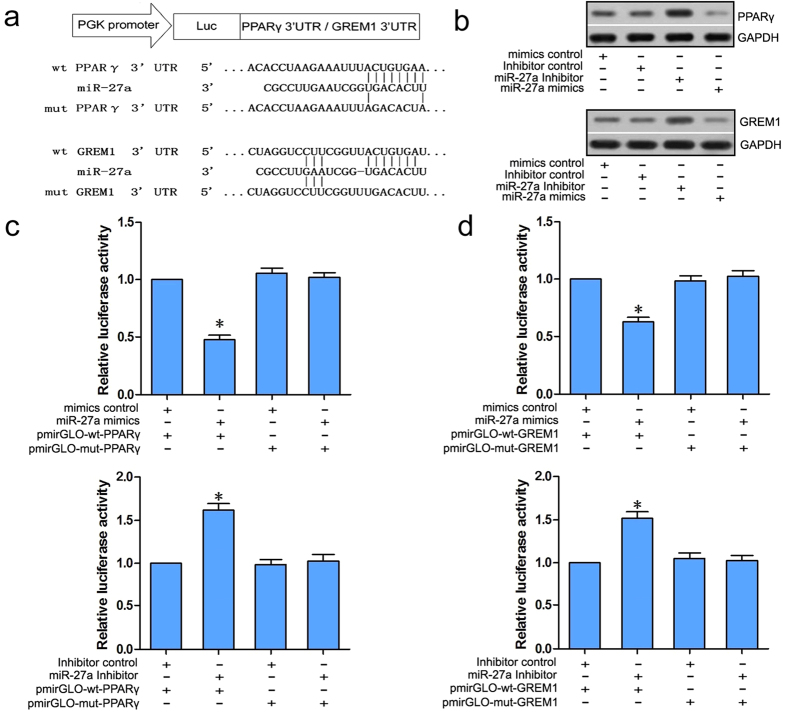
PPARγ and GREM1 were direct targets of miRNA-27a. (**a**) PPARγ and GREM1 share a matching 3′UTR sequence that targets the seed region of miR-27a. The sequences are presented. (**b**) Western blots showed the down-regulation of PPAR**γ** and GREM1 in the miR-27a mimics group and up-regulation of PPAR**γ** and GREM1 in the miR-27a inhibitor group compared with the mimics or inhibitor control groups. (**c, d**) The dual luciferase assay showed significantly reduced luciferase activity in BMSCs that were co-transfected with the miR-27a mimics and pmirGLO-wt-PPARγ compared with cells co-transfected with miR-27a mimics and pmirGLO-mt-PPARγ, and significantly enhanced luciferase activity was observed in BMSCs that were co-transfected with miR-27a inhibitor and pmirGLO-wt-PPARγ compared with the cells that were co-transfected with the miR-27a inhibitor and pmirGLO-mt-PPARγ. A similar result was observed in BMSCs that were co-transfected with the miR-27a mimics and pmirGLO-wt-GREM1 or the cells that were co-transfected with the miR-27a inhibitor and pmirGLO-wt-GREM1. ^*^P < 0.05.

Bioinformatics analyses using TargetScan (http://www.targetscan.org/) and miRBase (http://www.mirbase.org/) found that the PPAR**γ** and GREM1 mRNAs both contain a matching 3′ untranslated region (UTR) sequence that targets the seed region of miR-27a; these sequences are presented in Fig. 2a. This result suggests that PPAR**γ** and GREM1 are potential target genes of miR-27a.

We next transfected rat BMSCs with miR-27a mimics, an miR-27a inhibitor, a mimics control or an inhibitor control to detect the interaction between miR-27a and PPAR**γ** and GREM1. Western blotting showed that PPAR**γ** and GREM1 were down-regulated in the miR-27a mimics group and up-regulated in the miR-27a inhibitor group compared with the relevant control groups (Fig. 2b).

Furthermore, dual luciferase assays showed significantly reduced luciferase activity in BMSCs that were co-transfected with the miR-27a mimics and pmirGLO-wt-PPARγ compared with the cells that were co-transfected with the miR-27a mimics and pmirGLO-mt-PPARγ. In addition, significantly enhanced luciferase activity was observed in BMSCs that were co-transfected with the miR-27a inhibitor and pmirGLO-wt-PPARγ compared with the cells that were co-transfected with the miR-27a inhibitor and pmirGLO-mt-PPARγ. A similar result was observed in BMSCs that were co-transfected with the miR-27a mimics and pmirGLO-wt-GREM1 or cells that were co-transfected with the miR-27a inhibitor and pmirGLO-wt-GREM1 (Fig. 2c,d). These results indicate that miR-27a can bind to the pmirGLO-wt-PPARγ and pmirGLO-wt-GREM1 mRNAs and negatively regulate luciferase activity, implying that PPARγ and GREM1 are potential targets of miR-27a.

### Adipogenic differentiation was enhanced, osteogenic differentiation was inhibited, and miR-27a was down-regulated in steroid-induced rat BMSCs

**Figure 3:**
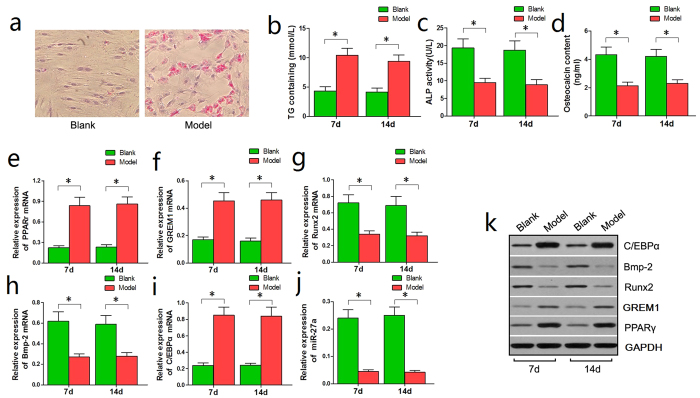
Adipogenic differentiation was enhanced, osteogenic differentiation was inhibited and miR-27a was down-regulated in steroid-induced rat BMSCs. (**a**) Oil red O staining detected more lipid droplets in BMSCs from the Model group compared with the Control group on day 14. (**b**) A significant increase in the TG content of BMSCs from the Model group was observed in comparison with the Control group on days 7 and 14. (**c,d**) Significant decreases in the ALP and OST contents in the Model group were observed in comparison with the Control group. (**e,f,i**) qRT-PCR showed that PPARγ, GREM1, and C/EBPα were significantly up-regulated in BMSCs from the Model group compared with the Control group. (**g,h,j**) qRT-PCR showed that Runx2, Bmp-2 and miR-27a were significantly down-regulated in BMSCs from the Model group compared with the Control group. (**k**) The western blotting results were consistent with the qRT-PCR results: PPARγ, GREM1, and C/EBPα were significantly up-regulated while Runx2 and Bmp-2 were significantly down-regulated in BMSCs from the Model group. *P < 0.05.

Next, the BMSCs from the Model group were continuously administered steroids for 14 days. The Blank group was used as the experimental control. On day 14 of steroid induction, oil red O staining showed increased lipid droplets in the BMSCs from the Model group compared to those from the Control group (Fig. 3a).

Enzyme-linked immunosorbent assays (ELISA) performed on days 7 and 14 revealed a significant increase in the triglyceride (TG) content of BMSCs from the Model group compared with the Control group (Fig. 3b), suggesting that adipogenesis was increased in the steroid-induced rat BMSCs. However, the alkaline phosphatase (ALP) content of BMSCs and the osteocalcin (OST) content in the culture medium were significantly reduced in the Model group compared with the Control group (Fig. 3c,d), suggesting that osteogenesis was inhibited in steroid-induced rat BMSCs.

We also examined the expression levels of adipogenesis-associated genes (PPARγ and C/EBPα) and osteogenesis-associated genes (GREM1, Runx2, and Bmp-2) to further examine the changes in adipogenic and osteogenic differentiation. The western blotting and qRT-PCR showed that PPARγ, GREM1, and C/EBPα were significantly up-regulated, whereas Runx2 and Bmp-2 were significantly down-regulated in BMSCs from the Model group compared with the Control group (Fig. 3e–k). These results support our findings of increased adipogenic differentiation and reduced osteogenic differentiation in steroid-induced rat BMSCs.

Moreover, the qRT-PCR results revealed that miR-27a was significantly down-regulated in BMSCs from the Model group compared with the Control group on day 7 and 14 (Fig. 3j). This result is in accordance with our miRNA microarray analysis and strongly suggests that miR-27a is involved in the biological processes of adipogenesis and osteogenesis in steroid-induced rat BMSCs.

### miRNA-27a up-regulation attenuated adipogenic differentiation and promoted osteogenic differentiation in steroid-induced rat BMSCs

**Figure 4:**
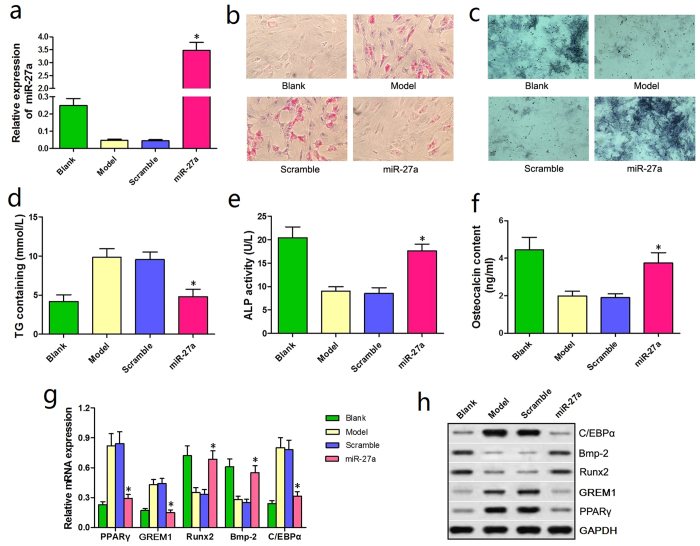
miRNA-27a up-regulation attenuated adipogenic differentiation and promoted osteogenic differentiation in steroid-induced rat BMSCs. (**a**) The qRT-PCR conducted at 24 h post-transfection showed that miR-27a expression was significantly increased in the miR-27a group compared with the Blank group. (**b**) Oil red O staining revealed fewer lipid droplets in BMSCs from the miR-27a group compared with the Model and Scramble groups. (**c**) ALP staining showed that a greater number of BMSCs were stained blue/purple in the miR-27a group than in the Model and Scramble groups. (**d**) A significantly reduction in the TG content was observed in the miR-27a group. (**e,f**) Significant increases in the ALP content of BMSCs and the OST content in the culture medium were observed in the miR-27a group. (**g**) The qRT-PCR results showed a significant reduction in the expression of adipogenesis-associated genes (PPARγ and C/EBPα). Furthermore, osteogenesis-associated genes were significantly and differentially expressed in BMSCs from the miR-27a group: GREM1 was down-regulated, whereas Runx2 and Bmp-2 were up-regulated. (**h**) The western blotting results were similar to the qRT-PCR results. *P < 0.05.

We generated rat BMSCs that overexpressed miR-27a (miR-27a group) to investigate the involvement of miR-27a in adipogenesis and osteogenesis. The qRT-PCR results confirmed successful transfection, with significantly higher miR-27a expression in the miR-27a group than in the Blank group (Fig. 4a).

On day 14 of steroid administration, oil red O staining showed that fewer lipid droplets were observed in BMSCs from the miR-27a group compared with the Model and Scramble groups (Fig. 4b). In addition, ALP staining showed an increased number of BMSCs that were stained blue/purple in the miR-27a group compared with the Model and Scramble groups (Fig. 4c).

ELISAs were performed to further verify the observed staining phenomena. On day 7 and 14, the TG contents were significantly reduced in BMSCs from the miR-27a group compared with the Model and Scramble groups (Fig. 4d). In contrast, the ALP content of rat BMSCs and the OST content in the culture medium were significantly increased in the miR-27a group compared with the Model and Scramble groups (Fig. 4e,f).

Correspondingly, the qRT-PCR and western blotting results showed that the expression of adipogenesis-associated genes (PPARγ and C/EBPα) was significantly reduced in BMSCs from the miR-27a group compared with the Model and the Scramble groups. Osteogenesis-associated genes (GREM1, Runx2, and Bmp-2) were significantly and differentially expressed: GREM1 was down-regulated, whereas Runx2 and Bmp-2 were up-regulated (Fig. 4g,h). These results suggested that miR-27a up-regulation effectively attenuated adipogenic differentiation and promoted osteogenic differentiation in steroid-induced rat BMSCs.

### Expression of PPARγ and GREM1 without 3′UTRs partially rescued the effects of miRNA-27a overexpression on adipogenic and osteogenic differentiation in steroid-induced rat BMSCs

**Figure 5:**
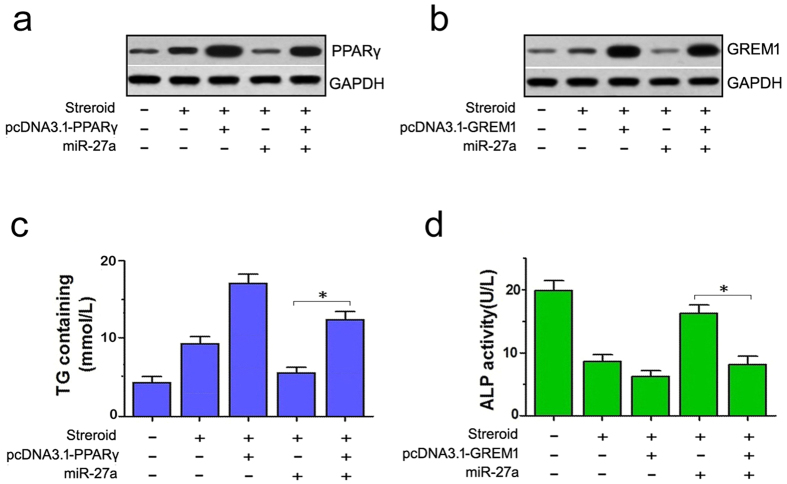
The expression of PPARγ and GREM1 without 3′UTRs rescued the effects of miRNA-27a overexpression on adipogenic and osteogenic differentiation in steroid-induced rat BMSCs. (**a,b**) Western blots showed the up-regulation of PPARγ and GREM1 in groups that were co-transfected with pcDNA3.1-PPARγ or pcDNA3.1-GREM1 (without 3′UTRs). (**c**) Higher TG levels were observed in steroid-treated BMSCs that were co-transfected with pcDNA3.1-PPARγ and miR-27a compared to the group that was transfected with miR-27a alone. (**d**) Lower ALP levels were observed in steroid-treated BMSCs that were co-transfected with pcDNA3.1-GREM1 and miR-27a compared to the group that was transfected with miR-27a alone. *P < 0.05.

We next co-transfected rat BMSCs with PPARγ or GREM1 expression vectors that lacked the 3′UTR to further confirm miR-27a attenuates adipogenic differentiation and promotes osteogenic differentiation by targeting PPARγ and GREM1. Transfections with these two vectors were successful and led to significantly up-regulated expression of PPARγ or GREM1, according to the western blotting results (Fig. 5a,b).

Next, on day 14 of steroid treatment, we tested the TG and ALP contents in the culture medium using ELISA. A significant increase in TG content was detected in the groups that were co-transfected with pcDNA3.1-PPARγ and miR-27a compared with the cells that were transfected with miR-27a alone, and this level was reduced in comparison to the group that was transfected with pcDNA3.1-PPARγ alone. Accordingly, reduced ALP content was detected in the groups that were co-transfected with pcDNA3.1-GREM1 and miR-27a compared with those that were transfected with miR-27a alone, and this level was higher in comparison to the group that was transfected with pcDNA3.1-GREM1 alone. These results showed that the expression of PPARγ and GREM1 without 3′UTRs could partially rescue the effects of miR-27a overexpression (Fig. 5c,d).

### miRNA-27a up-regulation had a stronger effect on the attenuation of adipogenic differentiation in steroid-induced rat BMSCs than si-PPARγ and si-GREM1

**Figure 6:**
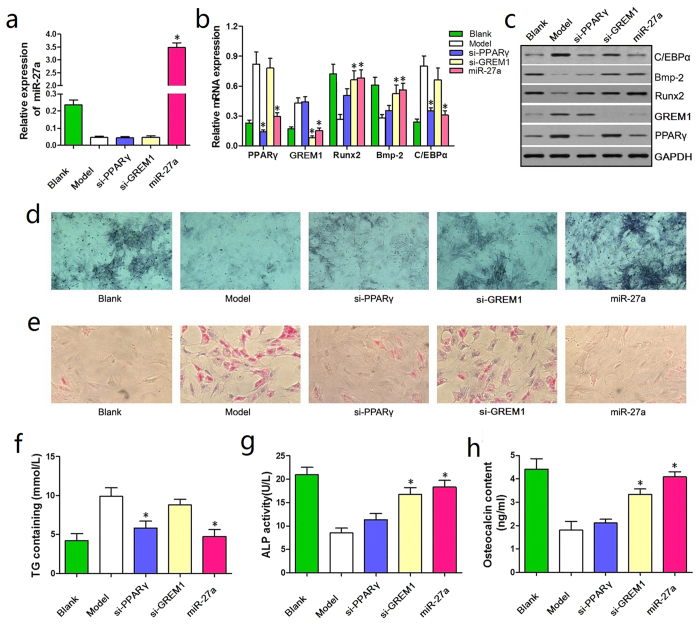
miRNA-27a up-regulation had a stronger effect on attenuating adipogenic differentiation in steroid-induced rat BMSCs than si-PPARγ and si-GREM1. (**a**) The qRT-PCR conducted at 24 h post-transfection demonstrated successful transfection. (**b**) Reduced PPARγ expression was observed in the miR-27a and si-PPARγ groups compared with the Model and si-GREM1 groups, and reduced expression of C/EBPα was observed in the miR-27a group. Furthermore, GREM1 was significantly down-regulated in the miR-27a and si-GREM1 groups, whereas Runx2 and Bmp-2 were significantly up-regulated in the miR-27a group compared with the Model, si-PPARγ and si-GREM1 groups. (**c**) The western blotting results were in accordance with the qRT-PCR results. (**d**) ALP staining revealed an increased number of BMSCs that were stained blue/purple in the miR-27a group than in the other groups. (**e**) Oil red O staining showed significantly fewer droplets in BMSCs from the miR-27a group compared with the other groups. (**f**) A significantly reduction in TG contents was observed in BMSCs from the miR-27a group compared with the Model, si-GREM1 and si-PPARγ groups. (**g,h**) Significant increases in ALP and OST content were observed in the miR-27a group compared with the Model, si-PPARγ and si-GREM1 groups. *P < 0.05.

We transfected rat BMSCs with miR-27a, si-PPARγ, and si-GREM1 to compare the efficacy of miR-27a, si-PPARγ, and si-GREM1 on adipogenic and osteogenic differentiation. Successful transfection was verified by qRT-PCR (Fig. 6a,b).

Next, we examined the genes and proteins associated with adipogenic and osteogenic differentiation. The western blotting and qRT-PCR results showed a significant reduction in PPARγ expression in the miR-27a and si-PPARγ groups compared with the Model and si-GREM1 groups, and lower expression of C/EBPα was observed in the miR-27a group than in the other groups. Furthermore, GREM1 was significantly down-regulated in the miR-27a and si-GREM1 groups, whereas Runx2 and Bmp-2 were significantly up-regulated in the miR-27a group compared with the Model, si-PPARγ and and si-GREM1 groups (Fig. 6c). Overall, miR-27a up-regulation had a stronger effect on simultaneously attenuating adipogenic differentiation and promoting osteogenic differentiation in steroid-induced rat BMSCs than si-PPARγ and si-GREM1.

On day 14 of steroid treatment, ALP staining revealed a greater number of BMSCs that were stained blue/purple in the miR-27a group in the Model, si-PPARγ and si-GREM1 groups (Fig. 5d). Furthermore, oil red O staining showed that there were fewer lipid droplets in BMSCs from the miR-27a group compared with the other groups (Fig. 6d,e).

ELISAs were then performed to confirm these results. Indeed, on day 7 and 14, the TG contents in BMSCs from the miR-27a group were significantly reduced compared with those in the Model group, si-GREM1 and si-PPARγ groups (Fig. 6f). However, the ALP content of rat BMSCs and the OST content in the culture medium were significantly increased in the miR-27a group compared with the other groups (Fig. 6g,h). These results suggest that compared with si-PPARγ and si-GREM1, miR-27 has a combinatorial effect on attenuating adipogenic differentiation and promoting osteogenic differentiation in steroid-induced rat BMSCs.

## Discussion

In recent years, emerging studies have shown that miRNAs are involved in adipogenesis and osteogenesis^21^,^22^,^23^. miRNAs are a class of small (18–24 nucleotides long), single-stranded, non-coding RNAs that are distributed in diverse tissues and participate in various biological activities in eukaryotes^24^. miRNAs down-regulate target genes by either promoting mRNA degradation or inhibiting their translation^25^ and play significant roles in cell proliferation and differentiation^26^. In particular, miRNAs such as miR-31, miR-34c, and miR-338–3p have been shown to be involved in regulating BMSC differentiation^27^,^28^,^29^. Moreover, altered miRNA expression has been identified in ONFH and other bone necrosis diseases^30^,^31^. Therefore, it is important to identify specific miRNAs and their targets that are involved in BMSC fate regulation and investigate the detailed mechanisms of steroid-induced ONFH to shed light on the disease pathogenesis and develop novel and effective therapeutic approaches.

Here, we identified 37 differentially expressed miRNAs in the femoral heads from the steroid-induced ONFH rat model. As detailedly described in the Methods part, we administered low dose of dexamethasone sodium phosphate every 24 hours for three times in total, which could successfully establish rat models of ONFH and lead to a lower mortality rate, compared with the traditional way of injecting a much higher dose on 7-day interval for three times in total^32^,^33^,^34^. Our previous studies prove that interval injection with a higher dose is more applicable in establishing animal models with a larger body figure such as rabbits. For rat models of ONFH, we recommend continuous injection with a low dose. Among the 37 differentiated expressed miRNAs, miR-27a was a down-regulated one, and subsequent bioinformatics and our previous studies showed that miR-27a was likely involved in the initiation of ONFH and had a close interaction with PPARγ and GREM1 as described before, this triggered us to further detect miR-27 and its role in ONFH. PPARγ is an adipogenic transcription factor that is expressed in the very early stage of adipogenesis^35^. This factor strongly promotes adipogenic differentiation and inhibits osteogenic differentiation^36^. Research has shown that steroids significantly up-regulate PPARγ expression in rodent and human BMSCs, leading to the time- and dose-dependent induction of adipogenic differentiation. The subsequent accumulation of adipocytes *in vivo* may result in an impeded blood supply, decreased osteogenic differentiation, insufficient bone regeneration, and finally, osteonecrosis^12^,^37^. Thus, PPARγ may be a key gene in the pathogenesis of steroid-induced ONFH. Indeed, we detected PPARγ up-regulation in the steroid-induced ONFH rat model and concomitant miR-27a down-regulation in the same femoral head tissue.

The other target identified was GREM1, an antagonist of bone morphogenesis protein-2 (Bmp-2), which can specifically bind to Bmp-2 and block its function^38^. Because Bmp-2 plays a vital role in the early osteogenic differentiation of BMSCs, inhibition of its function might cause decreased osteogenesis^39^. Indeed, studies have shown that GREM1 overexpression significantly suppresses osteocyte fate in cultured mouse BMSCs, whereas the administration of a GREM1-specific siRNA administration promotes osteogenesis^40^,^41^. Moreover, in GREM1 transgene mice, bone density is decreased by 20%, and the number of osteoblasts and trabecular bone are decreased by 70%^42^.

Because the differentiation of cultured BMSCs can simulate homeostasis of cell fate determination *in vivo*, we was further examined miR-27a function in BMSCs isolated from rats by transfecting BMSCs with miR-27a mimics using Lipofectamine™ 2000 (specified in the following Methods part). Since the miRNAs degrade in transiently transfected cells over time, 50 nM miRNAs are usually adopted as a working concentration, which can lead to an overexpression of the miRNA at the early stage after transfection. However, this method is well accepted by many researchers in miRNA study since it can detect miRNA function with more convenience and less expense^43^,^44^,^45^. Here, we found that miR-27a up-regulation enhanced osteogenic differentiation and suppressed adipogenic differentiation by inhibiting PPARγ and GREM1 in steroid-induced rat BMSCs. We can avoid the temporary overexpression with lentiviral transfection, a more ideal method to get more persuasive data, by modifying cell MOI and selecting the steadily expressing cells. This is an imperfection of our present study. Relevant adipogenesis- and osteogenesis-associated genes were examined to identify the corresponding biological behaviors. In addition to the functional investigation of miR-27a, we hypothesize that these genes (including Runx2, Bmp-2, and C/EBPα) may have latent interactions with PPARγ and GREM1 to regulate adipogenesis and osteogenesis. Indeed, the detailed regulatory pathways of these genes still require further investigation. Compared with the siRNAs targeting PPARγ and GREM1, miR-27a showed a stronger effect on comprehensively regulating adipogenic and osteogenic differentiation. These results show that by targeting both PPARγ and GREM1, miR-27a regulates BMSC differentiation in a combined manner and thus serves as a potent regulator of the balance between adipogenesis and osteogenesis.

In conclusion, we identified 9 up-regulated and 28 down-regulated miRNAs in the femoral heads of the steroid-induced ONFH rat model. In particular, miR-27a was down-regulated in steroid-induced ONFH. Moreover, miR-27a up-regulation could both inhibit adipogenesis and promote osteogenesis by directly targeting PPARγ and GREM1. Hence, we identified miR-27a as a probable key regulator of adipogenesis in steroid-induced BMSCs and a potential therapeutic target for ONFH treatment. However, further studies on the detailed mechanisms and feasibility are still required before miR-27a can be targeted for clinical applications.

## Methods

### Establishment of a steroid-induced ONFH rat model and relevant pathological examinations

Sprague-Dawley (SD) rats were purchased from Henan Experimental Animals Center (Zhengzhou, China). A total of 30 rats (15 male and 15 female) were randomized into two groups: the Model group and the Control group. The rats in the Model group were treated with dexamethasone sodium phosphate treatment via an intramuscular injection at a dose of 10 mg/kg for a total of three times with an interval of 24 hours. The rats in the Control group were administered normal saline. The rats were anesthetized by inhalation of sevoflurane when receiving the intramuscular injection. Four weeks later, three rats in each group were randomly sacrificed. The femoral heads were decalcified and then stained with HE. Successful construction of the animal model was defined by an empty pit, trabecular bone fracture, and fibrous structure hyperplasia. This study was approved by the Animal Experimentation Ethics Committee of Zhengzhou University and all involved methods were carried out in accordance with the approved guidelines.

### miRNA microarray analysis

Three randomly selected rats from each of the Model and Control groups were sacrificed 5 weeks after modeling. The femoral heads were obtained, immediately frozen in liquid nitrogen, and shipped on dry ice for further miRNA microarray analysis at Shanghai Biotechnology Co., Ltd. (Biotechnology Co., Ltd, Shanghai, China).

### Isolation and culture of BMSCs

BMSCs were isolated from the tibias and fibulas of 2- to 3-week-old healthy SD rats. Sevoflurane was inhaled to induce anesthesia. The bone marrow was washed and cultured in α-minimal essential medium (α-MEM, Gibco, CA, USA) containing 15% fetal bovine serum (FBS; Gibco, CA, USA), 100 U/mL penicillin, 100 μg/mL streptomycin, 1 mmol/L L-glutamine, and 20 mmol/L Hepes 20. The cells were seeded into culture vessels at a density of 6 × 10^6^ cells/mL and cultured in a humidified incubator at 37 °C with 5% CO_2_. The medium was replaced after 48 h of culture. The adherent cells were recognized as BMSCs. After two cell culture passages, BMSCs with good cell morphology and growth were selected for surface markers analysis (including CD29, CD34, CD44, CD45, and CD105) using a FACScan flow cytometer (BD Biosciences, CA, USA).

### RNA oligoribonucleotides and cell transfection

The RNA oligoribonucleotides (miR-27a mimics, miR-27a inhibitor, mimics control, inhibitor control, si-PPARγ, and si-GREM1) used in this study were synthesized by Shanghai GenePharma Co., Ltd. (GenePharma Co., Ltd, Shanghai, China). Prior to transfection, BMSCs (2 × 10^6^ cells/mL) that had been isolated and previously cultured were seeded (2 × 10^6^ cells/mL) into 6-well plates and grown until they were 60–80% confluent. The cells were then transfected with the RNA oligoribonucleotides using Lipofectamine™ 2000 (Invitrogen, Carlsbad, CA, USA), according to the manufacturer’s instructions. The cells were then seeded into 6-well plates. The transfection efficiency was assessed by qRT-PCR at 24 h post-transfection.

### Steroid induction

BMSCs (including transfected and non-transfected cells) were seeded into 6-well plates at a density of 1 × 10^6^ cells/mL at 24 h post-transfection. The medium was replaced every 2 days and 10^−7^ mol/L dexamethasone was added. A microscope was used to observe the growth conditions of the BMSCs, and the changes were thoroughly recorded.

### Total RNA extraction and qRT-PCR

Total RNA was extracted using a total RNA extraction kit (Omega, Norcross, GA, USA), according to the manufacturer’s instructions. cDNAs were synthesized using the RevertAid First Strand cDNA kit (Thermo Fisher Scientific, Waltham, MA, USA). The miR-27a expression levels were assessed with qRT-PCR using an ABI 7500 thermal cycler and a High-Specificity miR-27a qRT-PCR Detection Kit (Stratagene Corp, La Jolla, CA, USA), according to the manufacturer’s instructions. U6 small nuclear RNA (snRNA) was used as the internal reference for normalization of the miR-27a levels. The results were calculated and measured using the 2^−∆Ct^ method.

The expression levels of mRNAs including PPARγ, GREM1, Runx2, Bmp-2, and C/EBPα were verified by qRT-PCR using SYBR Green I (Takara, Dalian, China), according to the manufacturer’s instructions. β-actin was used as the internal reference to normalize the expression levels. Each experiment was performed in triplicate.

### Western blotting

Proteins were extracted from the BMSCs from each group using RIPA buffer, according to the manufacturer’s instructions. A BCA Protein Assay kit (Beyotime, Beijing, China) was used to determine the protein concentrations. Equal amounts of proteins were loaded onto sodium dodecyl sulfate-polyacrylamide gels (SDS-PAGE) and transferred to polyvinylidene difluoride (PVDF) membranes. The membranes were incubated with the primary antibodies overnight at 4 °C (1:300 dilution, monoclonal mouse anti-PPARγ, anti-GREM1, anti-Runx2, anti-Bmp-2, or anti-C/EBPα; Santa Cruz Biotechnology, Dallas, TX, USA), followed by incubation with a secondary antibody (1:3,000 dilution, horseradish peroxidase-conjugated rabbit anti-mouse IgG, Santa Cruz Biotechnology, Santa Cruz, USA) at 37 °C for 1 h. The blots were examined using a chemiluminescence detection kit (Amersham Pharmacia Biotech, Piscataway, NJ, USA). An antibody against GAPDH (Santa Cruz Biotechnology, Santa Cruz, USA) served as an internal reference.

### Oil red O staining

Cell cultures from the Model group, Scramble group, and miR-27a group were terminated on day 14 after dexamethasone administration. The Blank group was established as an experimental control. The BMSCs from the four groups were gently washed twice with PBS, fixed with 15% neutral formalin for 1 h, and stained with oil red O solution (Jiancheng Biotechnology, Nanjing, China) for 15 min at room temperature. Cell morphology and the staining patterns were then examined using a microscope.

### ALP staining

The ALP staining (Jiancheng Biotechnology, Nanjing, China) was performed on day 14 after dexamethasone treatment. The BMSCs from each group were washed 2–3 times with PBS and fixed with 4% formalin for 1 min. The stationary liquid was then removed followed by two TBST washes and ALP staining for 20 min at room temperature in the dark. Finally, the stained BMSCs were preserved in PBS. The results were examined using a microscope.

### Determination of cellular TG, ALP, and OST contents in the culture medium

On day 7 and day 14 of steroid induction, the cellular TG, ALP, and OST contents in the culture medium were determined using the corresponding ELISA kits (R&D Systems, Minneapolis, MN, USA). The BMSCs from each group were harvested at a density of approximately 1 × 10^6^ cells/mL. For TG determination, cell pellets were obtained after centrifugation at 1000 r/min for 10 min and were then gently washed twice with PBS. Next, 300 μl of a 1% Triton-X-100 cell lysis solution was added and the cell suspensions were incubated at 37 °C for 5 min. A Blank group was established for comparison. The absorbance values were determined at a wavelength of 500 nm (OD_500_). The ALP and OST contents were determined similarly, according to the corresponding instructions, except that the supernatant acquired after centrifugation was used for the ALP measurements and the BMSC culture medium was used for the OST measurements. Each experiment was repeated three times, and the relevant OD values determined.

### Dual luciferase assay

The bioinformatics analysis suggested that PPARγ and GREM1 were potential target genes of miR-27a. The 3′UTR fragments of both PPARγ and GREM1 were PCR amplified from the human genome. The corresponding mutant fragments were amplified by overlap extension PCR. The primers used are as follows: PPARγ, Forward 5′ GACGAGCTCCAGAAAAGTCCCAGTCGCTGACAAAG 3′, Reverse 5′ CATCTCGAGTA TTAAAAGTAAATTGTAAATGTATC 3′, Mutant-Reverse 5′ATGCTTTTTAGTGTCTAAAT TTCTTAGGTGTCAGATTTTTTTCCC 3′, Mutant-Forward 5′ AAGAAATTTAGACACTA AAAAGCATTTAAAAACAAAAAGTTTTAG 3′; GREM1, Forward 5′ GAAGAGCTCAA GCCACACACCAGATAAGTCTGAGT 3′, Reverse 5′ CATCTCGAGTAACATCTCTTCAT ATGTGACAAGAC 3′, Mutant-Reverse 5′ ACATTCGAAAAGTGTCAAACCGAAGGACC TAGAATTCCTAATTAC 3′, Mutant-Forward 5′ GTCCTTCGGTTTGACACTTTTCGAATG TTTTCTTTCTCTGTTTTA 3′.

Next, four different recombinant vectors were constructed: pmirGLO-wt-PPARγ, pmirGLO-mt-PPARγ, pmirGLO-wt-GREM1, and pmirGLO-wt-GREM1. Using Lipofectamine™ 2000 (Invitrogen, Carlsbad, CA, USA), the BMSCs were co-transfected with the miRNAs (miR-27a mimics, miR-27a inhibitor, mimics control or inhibitor control) and reporter vectors (wild-type or mutant-type). A Dual-Luciferase Assay kit (Promega, Madison, WI, USA) was used to detect luciferase activity according to the manufacturer’s instructions.

### Rescue assays

The Open Reading Frames (ORFs) fragments of PPARγ and GREM1 genes without 3′UTRs were amplified by PCR, and two recombinant vectors were constructed using pcDNA 3.1 plasmid (Invitrogen, Carlsbad, CA, USA): pcDNA3.1-PPARγ and pcDNA3.1-GREM1. Steroid-treated BMSCs were then separately transfected with pcDNA3.1-PPARγ, pcDNA3.1-GREM1, or the miR-27a mimics, or co-transfected with the expression vectors and miR-27a mimics. Blank groups without steroid treatment were used as controls. Western blotting was then used to examine PPARγ and GREM1 expression. Seven days later, the cellular TG and ALP contents in the medium were deteremined by ELISA to evaluate the altered differentiation of BMSCs.

### Statistical analyses

SPSS 17.0 (SPSS Inc., Chicago, IL, USA) was used for the statistical analyses. The data were presented as means ± standard deviation (SD). One-way analysis of variance (ANOVA) and least significant difference (LSD) multiple comparison tests were used to compare the differences between groups. Differences were regarded as statistically significant when *P *< 0.05.

## Additional Information

**How to cite this article**: Gu, C. *et al*. miR-27a attenuates adipogenesis and promotes osteogenesis in steroid-induced rat BMSCs by targeting PPARγ and GREM1. *Sci. Rep.* **6**, 38491; doi: 10.1038/srep38491 (2016).

**Publisher's note:** Springer Nature remains neutral with regard to jurisdictional claims in published maps and institutional affiliations.

## Figures and Tables

**Table 1:** Differentially expressed miRNAs in the femoral heads of the steroid-induced ONFH rat model.

miRNAs	Regulation	Log FC	miRNAs	Regulation	Log FC
miR-140	up	2.981	let-7a-1	down	−3.814
miR-10b	up	3.014	miR-181b-1	down	−3.725
miR-451	up	3.252	miR-450a	down	−3.762
miR-127	up	3.367	miR-27a	down	−3.684
miR-136	up	3.518	miR-133a	down	−3.585
miR-182	up	3.602	miR-24-1	down	−3.507
miR-486	up	4.257	miR-23a	down	−3.488
miR-144	up	4.401	miR-98	down	−3.471
miR-379	up	5.362	miR-19b-1	down	−3.427
miR-205	down	−6.515	miR-195	down	−3.401
miR-1247	down	−5.844	miR-30b	down	−3.349
miR-34a	down	−4.706	miR-20a	down	−3.341
miR-221	down	−4.501	miR-17-1	down	−3.317
miR-130b	down	−4.387	miR-301a	down	−3.284
miR-23b	down	−4.267	let-7e	down	−3.216
miR-214	down	−4.012	miR-150	down	−3.172
miR-203a	down	−4.007	miR-21	down	−3.094
miR-497	down	−3.974	miR-22	down	−3.052
miR-27b	down	−3.861			
